# High magnification optical imaging systems for the characterization of soft X-ray focii

**DOI:** 10.1107/S160057752500774X

**Published:** 2025-10-10

**Authors:** Andre Al Haddad, Antoine Sarracini, Kirsten Schnorr, Gregor Knopp, Juraj Krempasky, Christopher Arrell, Sven Augustin, Loïc Bassement, Katherine Brupbacher, Joan Vila-Comamala, Christian David, Uwe Flechsig, Rolf Follath, Zhaoheng Guo, Markus Herzog, Jonas Knurr, Csaba Lombosi, Eloisa Manetti, Suddhasattwa Mandal, Ana Sofia Morillo-Candas, Peng Qi, Boris V. Sorokin, Scott Stubbs, Zhibin Sun, Simon Christian Tiefenbacher, Jakub Vonka, Ulrich Hilmar Wagner, Xinhua Xie, Ningchen Yang, Hankai Zhang, Christoph Bostedt

**Affiliations:** a Paul-Scherrer Institute CH-5232Villigen PSI Switzerland; b LUXS Laboratory for Ultrafast X-ray Sciences, Institute of Chemical Sciences and Engineering École Polytechnique Fédérale de Lausanne (EPFL) CH-1015Lausanne Switzerland; Tohoku University, Japan

**Keywords:** soft X-rays, imaging, focus characterization, micrometric resolution, Talbot imaging

## Abstract

We present a series of novel X-ray imaging systems designed specifically for the soft X-ray energy range, optimized for operation in ultra-high-vacuum environments and compactness. These systems achieve micrometre-level spatial resolution with high collection efficiency of visible light by using high numerical aperture optics. Comprehensive characterization of the systems’ response was performed, including linearity assessments and X-ray sensitivity measurements, across X-ray photon densities ranging from 1 nJ m^−2^ to 10^−4^ nJ m^−2^. The imaging system was employed for caustic measurements to characterize the X-ray focal spot and to demonstrate its capabilities. Finally, grating interferometry was used to measure the wavefront distortion, yielding a pitch resolution as fine as 3.1 µm. These results underscore the system’s potential for high-resolution soft X-ray imaging and wavefront characterization applications.

## Abstract 2

We developed compact soft X-ray imaging systems optimized for ultra-high-vacuum environments, achieving micrometer-level spatial resolution with high collection efficiency. Characterization confirmed excellent sensitivity and linearity, with applications demonstrated in caustic measurements and grating interferometry for wavefront analysis.

## 1. Introduction

X-ray imaging detectors with micrometre to sub-micrometre spatial resolution typically employ direct camera detection, phosphor screens or scintillators. Direct camera systems are limited by pixel size (typically >5 µm) and the absorption efficiency of silicon in direct beams. In contrast, phosphor screens, despite achieving high resolution, are complex to fabricate and operate. The highest performance in the 5–100 keV range has been obtained using high-resolution detectors that couple single-crystal scintillators with optical magnification and CCD/CMOS cameras, enabling sub-micrometre resolution by relaying X-ray images through microscope objectives and lens–camera combinations (Stampanoni *et al.*, 2002 ▸; Koch *et al.*, 1998 ▸; Wang *et al.*, 2001 ▸). Scintillators are central to these systems, converting X-rays into visible light captured by imaging optics and transmitted to the camera. The achievable resolution depends on the scintillator material, thickness, substrate properties and optical quality. High-*Z* materials provide superior absorption efficiency, particularly in the hard X-ray regime (Stampanoni *et al.*, 2002 ▸; Koch *et al.*, 1998 ▸; Wang *et al.*, 2001 ▸), but thin films (as thin as ∼1 µm) required for maximal resolution introduce challenges such as substrate-induced aberrations, scattering and non-uniformity due to surface roughness (Martin & Koch, 2006 ▸; Wollesen *et al.*, 2022 ▸). The process of visible-light generation in the scintillation layer also affects the point spread function (PSF). Spatial resolution is primarily limited by photoelectric absorption, Rayleigh scattering and Compton scattering, with their relative contributions depending on photon energy and scintillator composition (Martin & Koch, 2006 ▸). Additional broadening of the PSF may arise from substrate luminescence, such as from Ce impurities in undoped YAG (Koch *et al.*, 1998 ▸; Martin *et al.*, 2017 ▸).

Koch *et al.* derived a formula to determine spatial resolution based on optical lens properties (Koch *et al.*, 1998 ▸; Martin & Koch, 2006 ▸). A lens and a camera focus on the object plane within the scintillator, while light from out-of-focus planes contributes to image degradation. Key factors affecting resolution include depth of focus, diffraction and spherical aberrations caused by the scintillator’s and substrate’s thickness. For utmost resolution, one would optimize for the highest numerical aperture (NA), use scintillators emitting at higher energies (lower wavelengths), reduce the substrate effects on aberration and secondary scattering, and use as thin as 1 µm scintillators (Wollesen *et al.*, 2022 ▸). Many studies report spatial resolutions of 1–2 µm, with advanced setups achieving resolutions below 1 µm (Martin & Koch, 2006 ▸; Wang *et al.*, 2001 ▸; Stampanoni *et al.*, 2002 ▸; Kameshima *et al.*, 2019 ▸).

This study aims to optimize conditions for achieving the highest spatial resolution in the soft X-ray regime (300 eV–1.8 keV). Operation in this energy range presents additional challenges due to the need for ultra-high vacuum (UHV), which complicates system design and implementation. In-vacuum cameras, while available, are expensive, difficult to operate, and limit both flexibility and spatial resolution.

The soft X-ray regime, however, offers distinct advantages. The high absorption cross section confines X-ray penetration to the first few hundred nanometres of the scintillator (*e.g.* ∼260 nm at 500 eV and ∼1 µm at 1.8 keV in Ce:YAG), enabling efficient dose deposition and limiting the visible-light generation region. Even with scintillators thicker than 1 µm, emission is constrained by this penetration depth. Within this range, the photoelectric effect dominates, generating short-range free electrons and minimizing lateral blurring.

These favorable properties can also introduce limitations. High absorption may cause rapid saturation and nonlinear visible-light generation, particularly with femtosecond X-ray pulses from free-electron lasers (FELs). Because the X-ray dose is deposited within femtoseconds—much shorter than the scintillator relaxation time (tens of nanoseconds) (Kirm *et al.*, 2000 ▸)—saturation thresholds of 25–38 J cm^−3^ have been reported (Krzywinski *et al.*, 2017 ▸).

Optical constraints in soft X-ray imaging essentially mirror those in the hard X-ray regime, with the added requirement of UHV operation (typically 10^−7^–10^−10^ mbar). The imaging system must be fully compatible with such conditions.

In this work, we present three systems designed for the soft X-ray range and UHV operation, applied to X-ray focus characterization and imaging. Detailed assessments of visible-light linearity, spatial resolution and uniformity demonstrate their potential for a broad range of advanced imaging applications.

## 2. Experimental setup

### 2.1. Concept

While similar concepts of the system have been explored in the hard X-ray regime, our work presents a novel extension and refinement tailored for optimal performance in the soft X-ray regime. This advancement builds upon established research, offering new insights and capabilities in a previously less-explored domain.

As outlined in the *Introduction*[Sec sec1], the central component of the system is the scintillator screen. For all experiments described herein, we utilized a scintillator comprising a 5 µm-thick Ce:YAG crystal glued to a 170 µm-thick antireflection-coated quartz substrate (Crytur). The scintillator thickness does not affect the resolution, as the penetration depth of the soft X-ray beam is confined to sub-micrometre scales. The visible light emitted by the scintillator is imaged using an infinity-corrected, high-magnification objective lens or an aspheric lens. Hence, the image propagation distance does not influence the quality of the final image.

The generated image is transmitted out of the vacuum chamber using a folding mirror through a vacuum viewport. Subsequently, it is focused using a 200 mm field lens onto a CCD or CMOS camera. Since the camera is located outside the vacuum, its selection can be adapted to suit various experimental needs, such as low noise, high quantum efficiency or high frame rate.

This study examines three distinct system geometries, each optimized for specific objectives: the first for achieving high spatial resolution, the second for compatibility with UHV environments, and the third for compactness. All systems are designed for seamless integration into existing experimental setups, with the capability to be positioned within the X-ray beam interaction region for imaging and subsequently retracted to allow experimental workflows to resume. This design enables *in situ* beam diagnostics with minimal disruption to ongoing experiments.

### 2.2. Off-the-shelf objective-based imaging system

Standard microscopy objectives can be directly employed in systems with moderate vacuum requirements without any modifications. Tests with commercial objectives from manufacturers like Nikon, Mitutoyo and Olympus demonstrated compatibility with vacuum pressures as low as approximately 1 × 10^−7^mbar.

**Figure 1:**
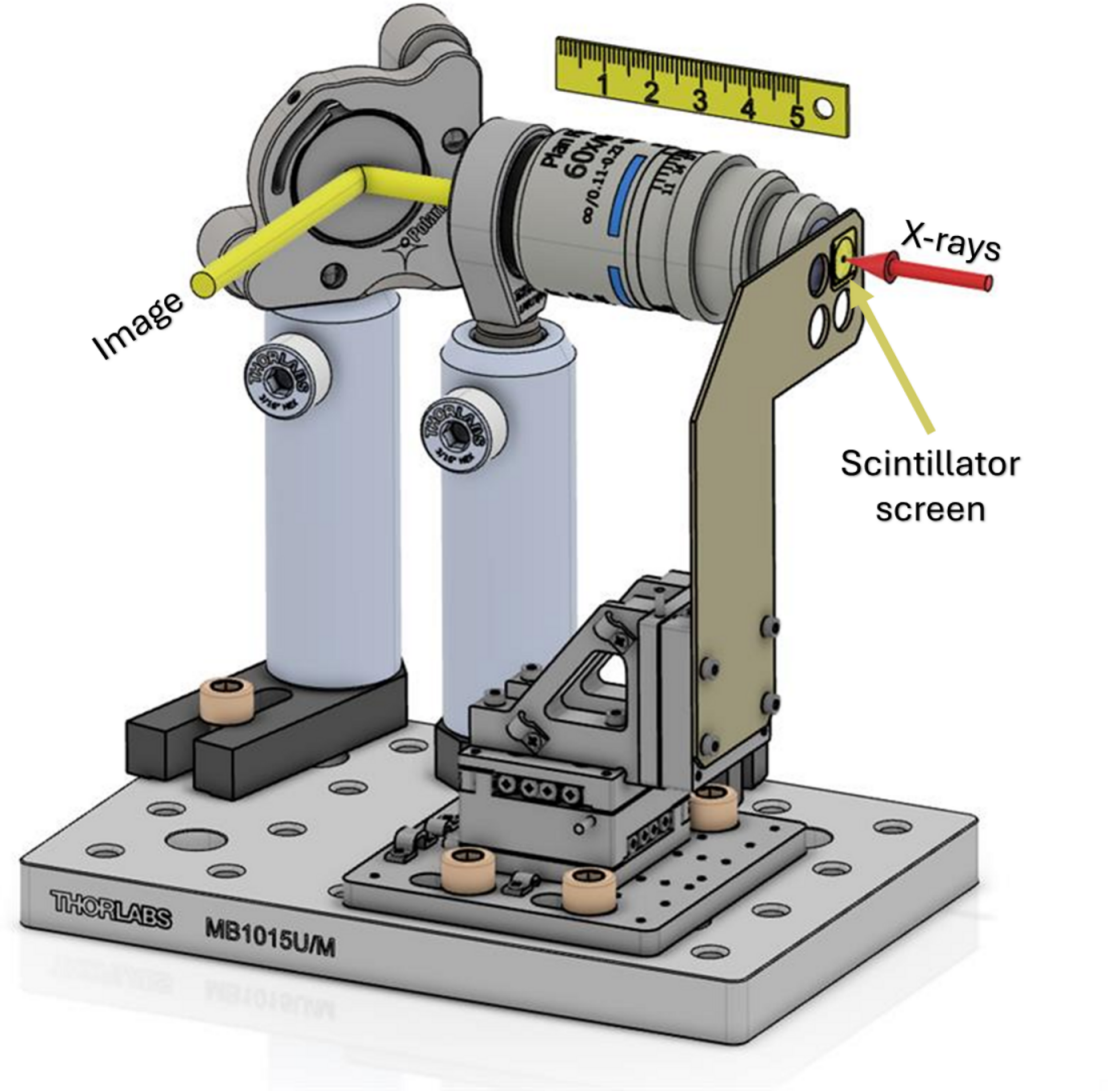
CAD-model of the imaging system, featuring an off-the-shelf objective, *XYZ* motion for precise scintillator positioning, and a mirror that directs the image (in yellow) outside the vacuum chamber. A field lens and camera are placed outside the vacuum to capture the image. The in-vacuum assembly is mounted on a movable stage, enabling quick insertion or removal from the beam path.

The system is depicted in Fig. 1 ▸. The scintillator screen was mounted on a three-axis (*XYZ*) SmarAct (XYZ-CLS32:32) motion system with nanometric precision, which allowed its front surface (the side exposed to X-rays) to be accurately positioned in the focal plane of the objective lens. This study presents results obtained with the Nikon N60X-PF objective; however, successful measurements have been performed using other lenses. This objective features a high NA of 0.85 and a working distance of 0.3–0.4 mm, adjustable using a collar ring. The high NA provides superior resolution and facilitates efficient light collection, enhancing the sensitivity of the measurements. The depth of field is ∼400 nm, which matches the mean penetration depth of soft X-rays.

The infinity-corrected image was directed to a vacuum viewport within the chamber using a 1-inch folding mirror. A 200 mm field lens was used outside the vacuum to focus the image onto the camera. The alignment of the mirror and field lens is crucial, as it can result in spatial resolution loss and image aberrations. A PCO.edge 5.5 CMOS camera recorded the images, offering a chip with 2560 × 2160 pixels with a 6.5 µm × 6.5 µm pixel size capable of running at 100 Hz repetition rate, matching the Swiss Free Electron Laser (SwissFEL) repetition rate. The camera was chosen for its low readout noise and ability to capture images of single X-ray pulses. This arrangement provided a field of view (FOV) of 160 µm × 200 µm with ∼0.083 µm per pixel sampling with a magnification of 60× at the camera with a theoretical Rayleigh resolution of 0.4 µm (at 550 nm).

### 2.3. Ultra-high-vacuum system

Standard commercial objectives are unsuitable for UHV conditions due to their construction materials, including adhesives that outgas and compromise vacuum quality. Reflective objectives, however, are well suited for UHV environments because they feature a monolithic design with little to no adhesives, significantly reducing outgassing risks. Such reflective objectives consist only of two mirrors in the Schwarzschild configuration, similar to an inverse Cassegrain system in a telescope with a primary concave mirror and a secondary convex mirror for collimation (Alaruri, 2018 ▸).

A custom reflective microscope objective, the Thorlabs LMM40X-P01, with NA = 0.5, was tested under UHV conditions. To ensure UHV compatibility, no paint or markings were applied to its casing during production. Using this lens, vacuum pressures as low as 2 × 10^−9^ mbar were achieved. The scintillator screen was aligned in the focal plane before installation in the vacuum system. For in-vacuum alignment, a compact piezo stage can be integrated to adjust the scintillator along the optical axis (X-ray propagation axis), enabling precise image focusing. A 1/4" folding mirror was employed to direct the infinity-corrected image toward the vacuum viewport. This system is similar to the one developed by Lame *et al.* at ESRF, but with slightly higher NA and independent positioning of the scintillator screen (Lame *et al.*, 2003 ▸). Coupled with a 200 mm lens and a Basler acA1920-25gm camera, this arrangement provided a FOV of 150 µm × 190 µm with ∼0.06 µm per pixel sampling with a magnification of 40× at the camera with a theoretical Rayleigh resolution of 0.7 µm (at 550 nm).

**Figure 2:**
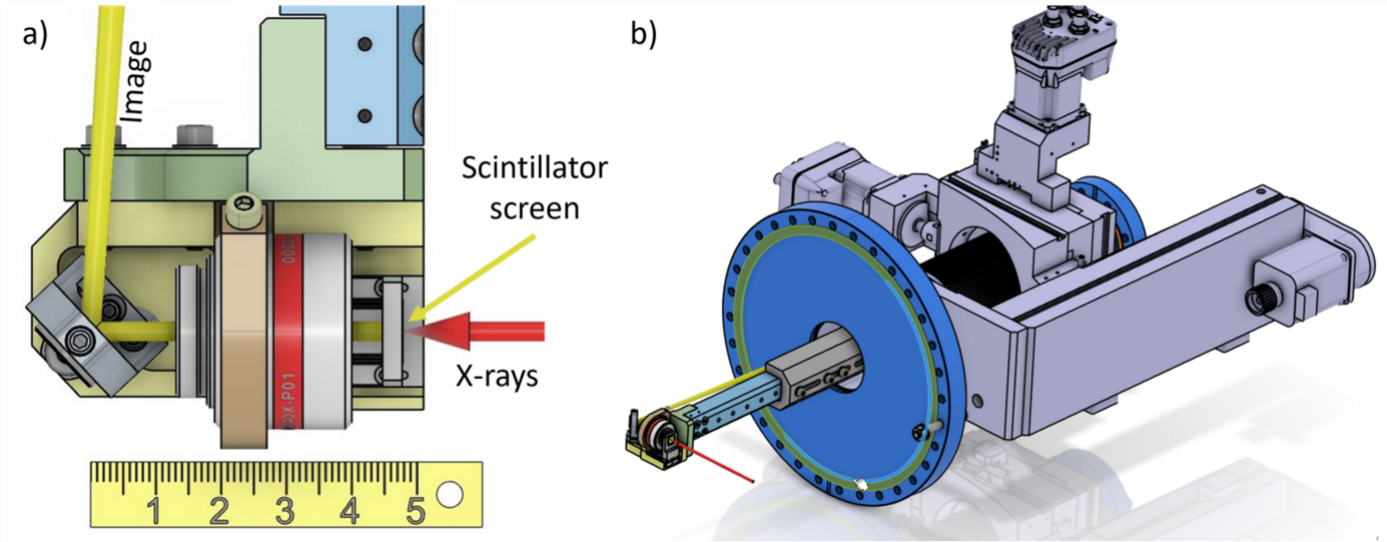
CAD-drawing of a compact imaging system utilizing a reflective objective. (*a*) Top view showing the incoming X-ray beam (in red), the scintillator, the reflective microscope, and a mirror directing the image outside the chamber. A linear motion stage aligns the scintillator at the focal plane of the objective. (*b*) 3D view of the system mounted on a three-axis manipulator. The image travels through the manipulator and exits via a window, where a field lens and camera are positioned. The manipulator enables precise placement of the entire system at the X-ray interaction point and allows for its removal to accommodate the main experiments.

The system, presented in Fig. 2 ▸, has compact dimensions of 60 mm × 40 mm × 50 mm and is mounted on a U-shaped rod to guide the optical image to the center of the DN-40 viewport. The rod was sanded to minimize scattering that could interfere with the camera. A magnetic shield was also designed to enclose the microscope, allowing X-rays to reach the scintillator while the optical image exited the chamber. This magnetic shield is essential for reliable operation in the vicinity of electron or ion spectrometers. The imaging system was mounted on a three-axis motion system, allowing for insertion and removal from the experimental interaction region.

### 2.4. Compact system

**Figure 3:**
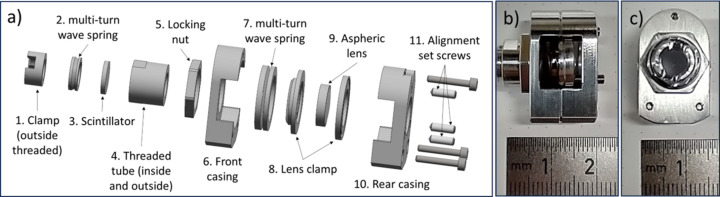
(*a*) An exploded view showing the individual components. The scintillator screen is housed in the threaded tube (4) and fixed in position using the clamp and spring (1, 2). The threaded tube (4) is then mounted into the front casing (6) and locked in position. By rotating (4), the distance of the scintillator relative to the casing and lens could be adjusted and locked. The aspheric lens is clamped between two metal rings and pushed towards the front casing by a spring (6, 7). The rear casing (10) holds the system together under stress. The alignment set screws (11) allow the adjustment of the lens angle and position relative to the scintillator screen. Panels (*b*) and (*c*) show photographs of the assembled system with a ruler for reference.

In specific scenarios, spatial constraints precede imaging considerations, as larger systems spanning several centimetres are not feasible to introduce into an operational system. Yet, spatial resolution of several micrometres is still desirable. To address this, we designed an ultra-compact system compatible with UHV conditions and capable of withstanding high temperatures during chamber bake-outs. The design is conceptually similar to previously presented systems but it omits the folding optic and replaces the large objective with an aspheric lens for enhanced compactness. A 5 mm-diameter scintillator screen is mounted using a threaded holder, enabling precise positioning relative to the lens. An aspheric lens is secured inside the holder using a steel multi-turn wave spring chosen for its uniform pressure distribution. Opposite the lens, three set screws provide fine adjustments to the lens’s angle and position. An exploded CAD-view and photographs are presented in Fig. 3 ▸.

The aspheric lens features a 4 mm focal length and a 6 mm aperture, yielding an NA of 0.42. Lens alignment is performed outside the vacuum and then inserted into the chamber. Coupled with a 200 mm lens and a Basler ace acA1920-48gm 2/3" camera, this arrangement provided a FOV of 200 µm × 130 µm with ∼0.1 µm per pixel sampling with a magnification of 40× at the camera. Despite its compact size and inherent limitations, the system achieved a resolution of approximately 2 µm.

## 3. Results and discussion

This section will discuss the alignment procedure, characterization and response linearity of the imaging system described in Section 2.2. The described methods are not limited to this system and could be performed identically for the other systems. The measurements were performed at the Athos soft X-ray branch of the SwissFEL at the Maloja endstation (Follath *et al.*, 2019 ▸; Abela *et al.*, 2019 ▸). Linear and circularly polarized X-ray pulses were used with pulse lengths of <40 fs (root mean square) (Schmidt & Calvi, 2018 ▸).

### 3.1. Alignment and characterization

Due to the limited FOV of the optical system (160 µm × 200 µm), it is difficult to align the imaging system to the X-ray beam. When back-illuminating the microscope using a high-brightness lamp, the microscope objective focused a white light spot onto the scintillator. A long-range optical microscope mounted out of a vacuum was used to locate and observe the optical focal spot on the scintillator by imaging the back-illumination focus, hence finding the focal point of the imaging system. The discrepancy between the X-ray position and the focal spot is determined by illuminating the scintillator screen with X-rays. The chamber and optical systems are mounted on a movable frame, allowing the positioning of the chamber relative to the X-ray beam with sub-10 µm precision. This procedure permitted the microscope to move towards the X-ray beam. Optimizing the position was done as a final step using the X-ray luminescence, where the beam was centered on the FOV. The scintillator screen was adjusted along the optical axis in sub-micrometre steps to position its X-ray illuminated surface at the center of the depth of focus of the microscope objective.

**Figure 4:**
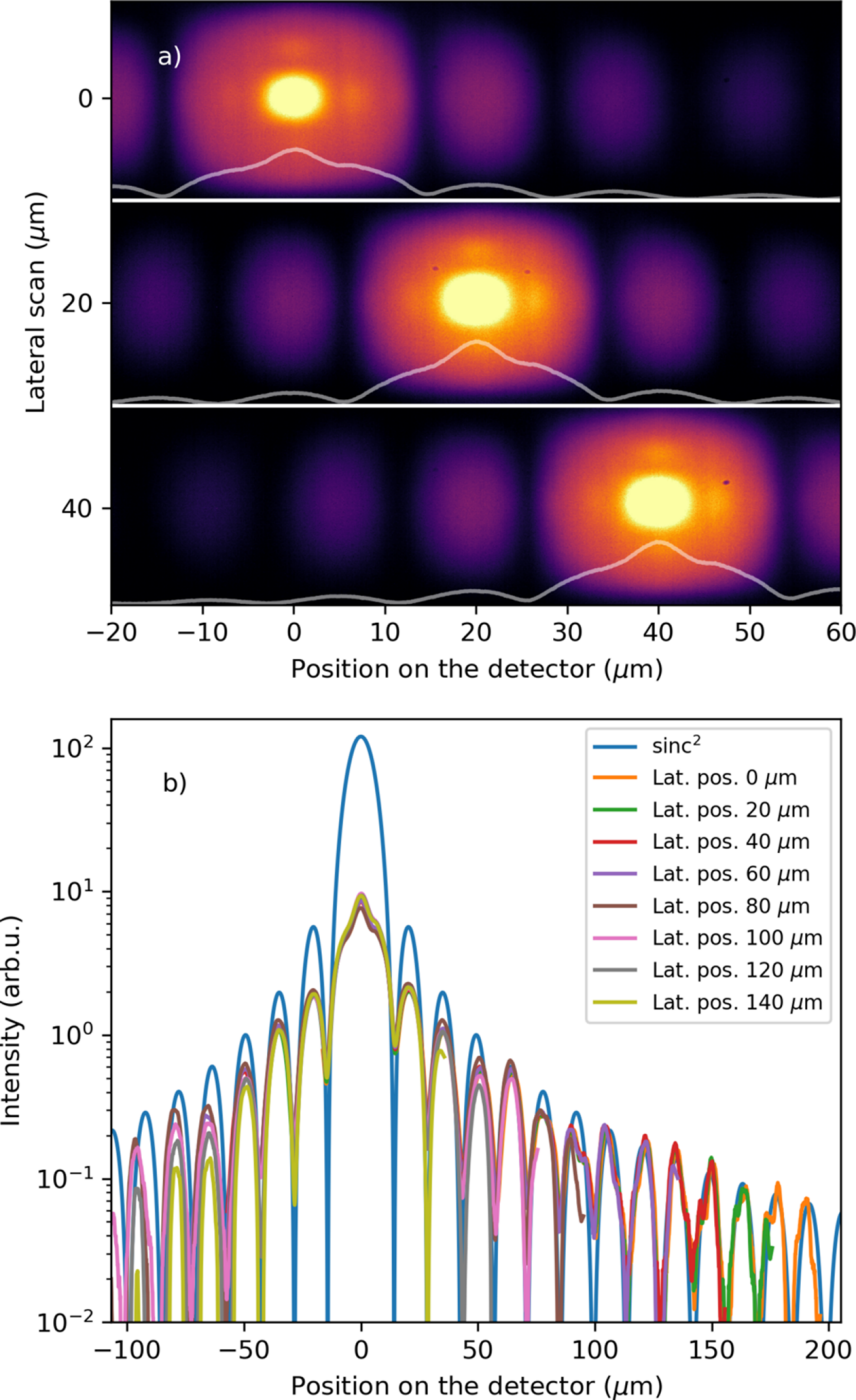
(*a*) Three diffraction patterns at 20 µm step in the lateral position of the imaging system relative to the X-ray beam. The white line is the projection along the vertical axis. (*b*) The diffraction pattern projection aligned at the lateral scan’s central peak from −40 µm to 40 µm with 20 µm step.

To calibrate and assess the aberrations of the imaging system, a set of slits was closed in the X-ray beam path to 0.3 mm × 0.3 mm at 8 m upstream of the KB optics. The diffraction pattern from the slits was focused by the KB optics onto the scintillator screen, as shown in Fig. 4 ▸(*a*). The diffraction pattern was swept through the image FOV by moving the detector in 20 µm steps in the lateral direction relative to the X-ray beam. By fitting the central peak to the table motion steps, a 12.38 pixels µm^−1^ calibration was found.

The diffraction pattern was then used to assess image aberration. In Fig. 4 ▸(*b*), the diffraction pattern projections are shifted to match the zero at the center of the patterns, showing the diffraction orders overlapping across the different scans. As we image the diffraction pattern in the far field, Fraunhofer diffraction equations describe the intensity profile observed for a rectangular aperture (Born & Wolf, 1999 ▸). Therefore, the intensity distribution in amplitude and space could be approximated by a sinc^2^(*x*) function. In Fig. 4 ▸(*b*), the blue curve represents an ‘ideal’ distribution function of the diffraction expected diffraction pattern.

First, we look at the peak positions of the diffraction orders across the images. On the positive side (right to the center) peak, the diffraction orders follow in position the ‘ideal’ distribution. On the contrary, the negative order (left to the center peak) positions deviate from the ‘ideal’ form. This effect is noticeable at orders lower than −3. As all the peaks behave similarly despite the image location within the FOV, this artifact could not be described by an aberration in the imaging system. This could only be explained by astigmatism by the KB optic in the horizontal direction, as it focuses the diffraction onto the scintillator.

Second, the intensity distribution evidently deviates from the expected distribution of rectangular slit diffraction. The sinc^2^(*x*) distribution has been scaled to match the higher orders of the diffraction. The central peak shows saturation and flattening of the peak. The saturation effect is reduced for the ±1 orders but is still evidently there. For the higher orders (to the right of the central peak), the peak intensity distribution follows the predicted intensity distribution. The negative orders (left of the center peak) show deviation in intensity relative to the different measurements. This effect is observed when the diffraction orders reach the edge of the image and vignetting dominates.

Finally, despite the central peak’s saturation, it shows a structured intensity distribution. A closer look at Fig. 4 ▸(*a*) indicates that the central peak has four hot spots in the center surrounded by four other peaks. The peaks are separated from the central peak by one-third of the distance to the first order. Therefore, these peaks are attributed to the diffraction from the third harmonic content of the X-ray beam.

### 3.2. Response linearity and sensitivity

In a FEL, the fundamental (first-order) beam is generated alongside second- and third-order harmonics, which typically have a few percent of the fundamental’s intensity. The second harmonic is often significantly weaker than the third. Additionally, the beam mode of the harmonics differs from that of the fundamental beam (Baumann *et al.*, 2023 ▸). This is true only when generating linearly polarized light. At the SwissFEL Athos branch, AppleX undulators can produce circularly polarized light, eliminating the third-harmonic contamination. To study the system’s response linearity, the beam intensity was variably attenuated using the gas-filled (N_2_) attenuator (Pradervand *et al.*, 2023 ▸; Schmidt & Calvi, 2018 ▸).

**Figure 5:**
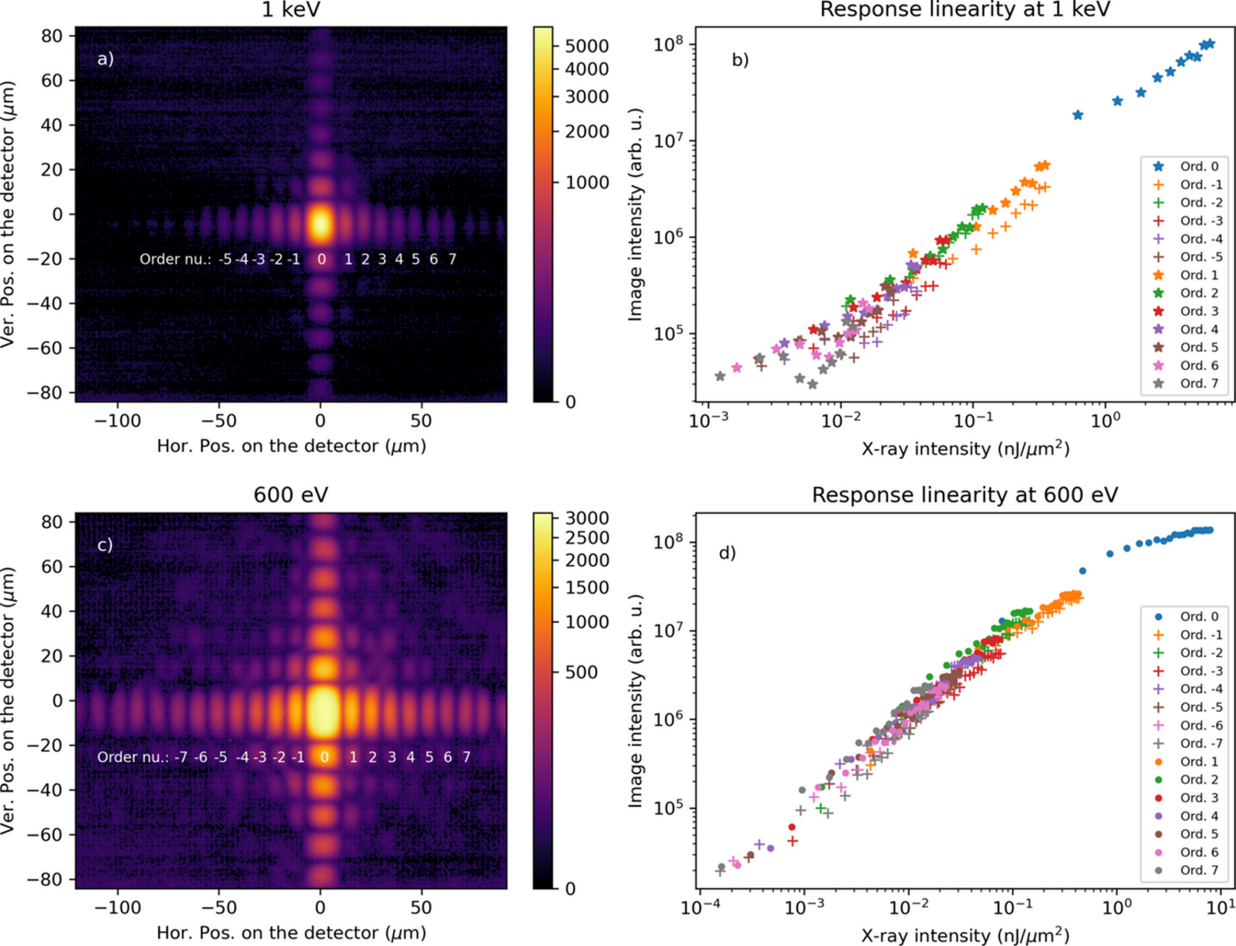
Panels (*a*) and (*c*) show diffraction patterns from 1 keV and 0.6 keV, respectively. The horizontal diffraction orders were used to calculate the system’s response, labeled in white in the image. Panels (*b*) and (*d*) show the image intensity versus local pulse energy density in the image. Positive and negative orders are labeled using a * and + markers, respectively.

Diffraction patterns were recorded at photon energies of 1 keV and 600 eV using slits to shape the beam. As shown in the structureless central peaks of the patterns [Figs. 5 ▸(*a*) and 5(*c*)], these data confirm the elimination of third-harmonic contamination. The X-ray beam was attenuated from 30 to 3 µJ, with 300 images of single X-ray pulses recorded for each attenuation level.

It is immediately apparent that the diffraction orders are magnified by roughly a factor of two along the vertical axis compared with the horizontal axis. This deviates from a diffraction pattern caused by a square slit. The primary distortion in the diffraction pattern arises from astigmatism in the KB mirror system, caused by the different effective source distances for the horizontal (3.85 m) and vertical (3.1 m) axes when the slits are closed. This offset is attributed to the distance between the centers of the two KB mirrors. This results in shifting the foci to 2.9 m and 5.4 m and producing a 53.6% magnification difference at the scintillator screen plane.

The Gaussian intensity distribution and pulse energy of the beam before the slits allowed estimation of the transmitted pulse energy. Based on the sinc^2^(*x*) intensity distribution, an energy density in nJ µm^−2^ was determined for each diffraction order. Horizontal diffraction orders [labeled in Figs. 5 ▸(*a*) and 5(*c*)] were used to evaluate the imaging system’s sensitivity and linearity. At 1 keV, a linear intensity response was observed across 10^−3^ nJ µm^−2^ to 10 nJ µm^−2^. However, at 600 eV, saturation effects became evident at 1 nJ µm^−2^, attributed to a higher local photon density caused by the shallower attenuation depth (0.22 µm versus 0.35 µm at 1 keV), leading to earlier saturation.

Krzywinski *et al.* reported a universal saturation of ∼33 J cm^−3^ for Cd:YAG exposed to sub-100 fs X-ray pulses ranging in energy from the EUV to hard X-rays. Considering a penetration depth of 600 eV X-ray photons (0.225 µm) and 1 nJ µm^−2^, a value of 4.4 × 10^3^ J cm^−3^ is obtained, two orders of magnitude higher (Krzywinski *et al.*, 2017 ▸). Despite this discrepancy, we observe a linear trend across several orders of magnitude in intensity. The detection limit of the measurement at 10^−4^ nJ µm^−2^ is due to the camera’s internal pixel noise. Extending the dynamic range to lower photon densities could be achieved with cooled CCD’s to reduce shot noise.

**Figure 6:**
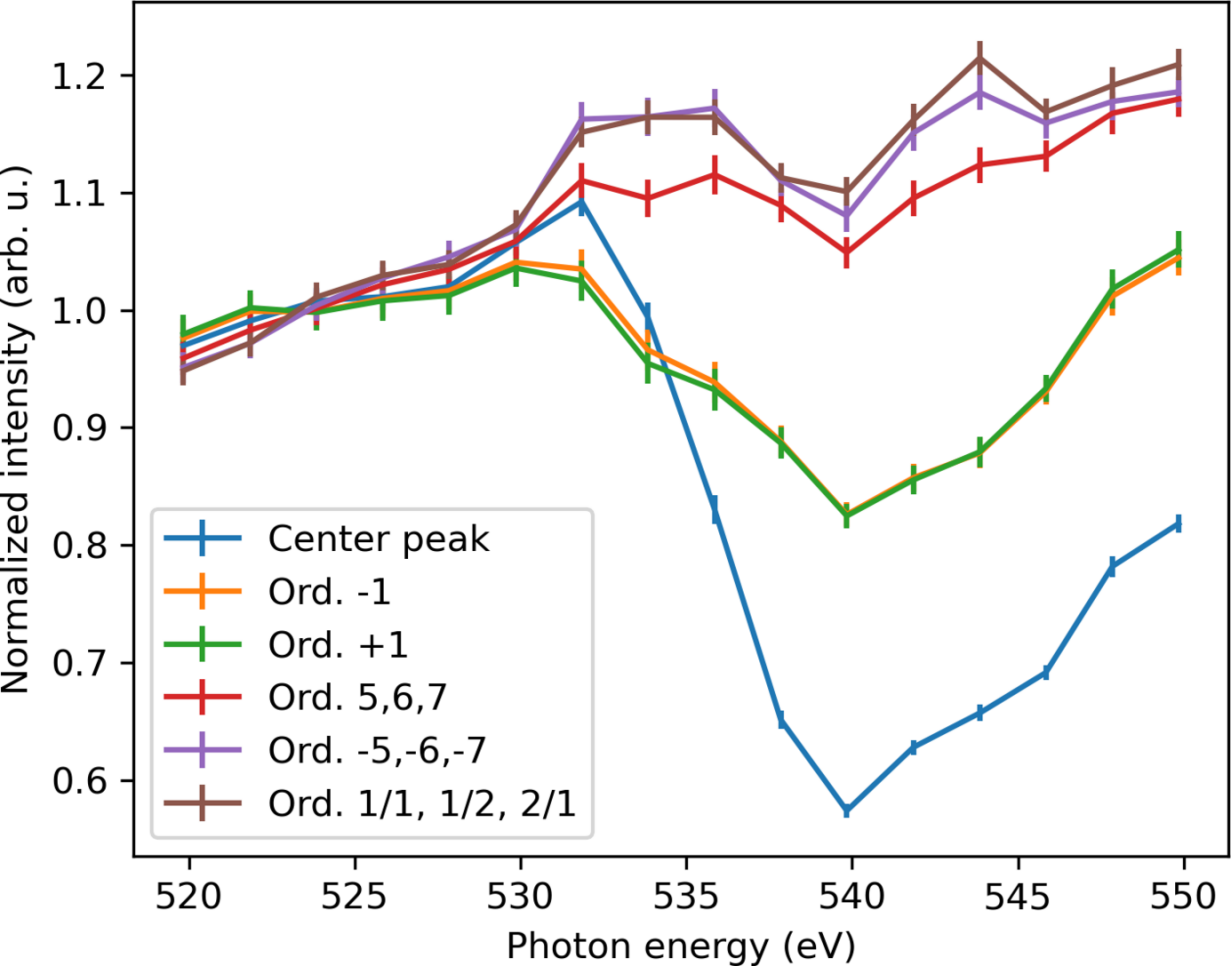
Intensity dependence of various diffraction orders to the photon energy around the O 1*s* absorption edge. Central peak and ±1 orders show dependence on the absorption edge, contrary to the other orders. Error bars represent standard error.

The photon energy was then scanned across the O 1*s* edge (520–550 eV) with 2 eV steps and an FEL bandwidth of 2.5 eV root mean square. Fig. 6 ▸ shows the normalized intensity response at 524 eV of various diffraction peaks versus photon energy. While the higher-order diffraction peaks maintained a linear trend with pulse energy, the central and ±1 diffraction orders exhibited a sudden reduction in intensity near the resonance. This behavior is explained by the significant decrease in penetration depth (from 0.27 to 0.18 µm) when crossing the absorption edge, corroborating the observed differences in linearity between the 600 eV and 1 keV datasets. Such a saturation effect would broaden and truncate any high-frequency features to be imaged.

### 3.3. Focus characterization and caustic measurements

Considering the spatial resolution and sensitivity, this imaging system is well suited to characterize the focal spot of a soft X-ray beam. In this section, we utilize our system to characterize the FEL-focused beam at 1.1 keV. A circularly polarized 1.1 keV beam of 48 µJ was attenuated by a series of carbon and silicon foils down to 0.13 nJ.

**Figure 7:**
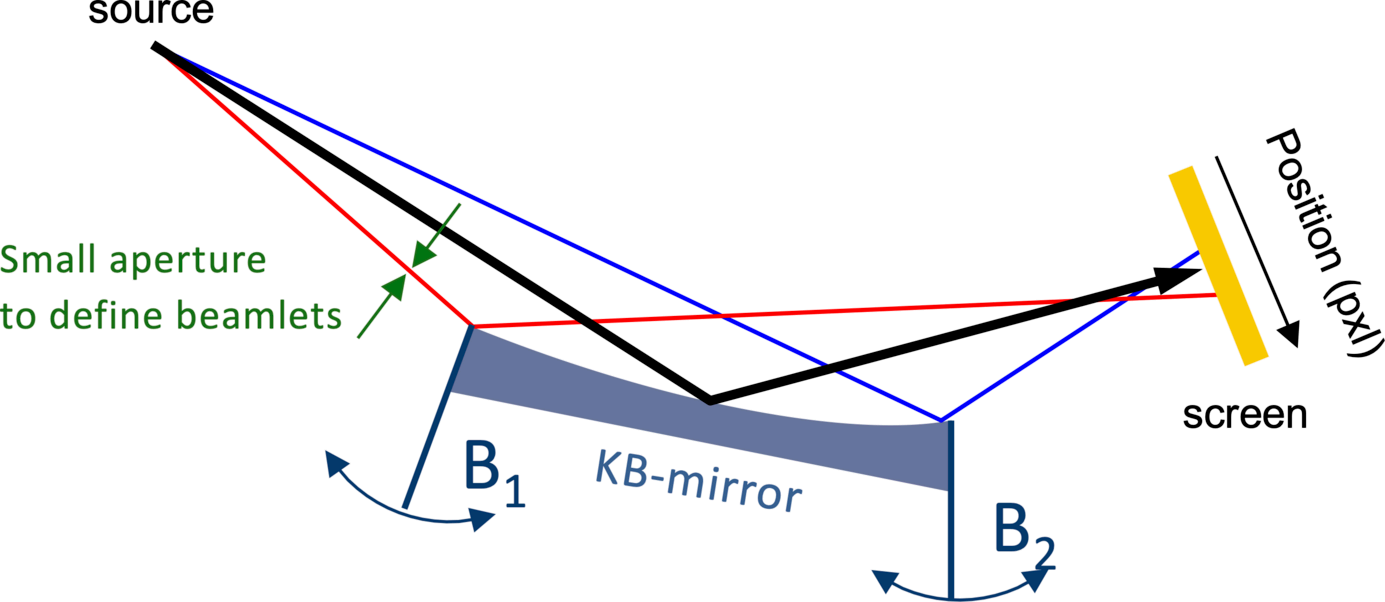
Optimizing the focal spot with the help of the bender motors B_1_ and B_2_. The beamlets are defined by the aperture indicated by green arrows and hit the mirror in the center (black) and its front (red) and rear (blue) parts.

A pair of flat but bendable mirrors in a KB configuration accomplishes the focusing of the FEL beam as shown in Fig. 7 ▸ (Follath *et al.*, 2019 ▸). Assuming a diffraction-limited source size of 50 µm FWHM and ideal optics, the spot size at the sample in pink beam mode should be approximately 0.5 µm × 0.7 µm (horizontal × vertical). Introducing torques at the ends of the mirror substrate can modify the surface profiles to form a stigmatic focal spot at any distance from infinity down to 1.5 m after the last mirror. Ahead of their installation into the beamline, the system is characterized offline in the metrology laboratory by scanning both motors over their full range and determining the induced surface profile with a long trace profiler. Although this already gives quite an accurate overview, the final tuning after installation and bakeout has to be done online with the FEL beam.

In the absence of a wavefront sensor, the optimization of the bending forces is done independently with the pencil beam technique for the vertically and horizontally focusing mirrors. A four-blade aperture in front of the KB-system defines three narrow beams with small diameters, called beamlets, that hit the mirror in its center and at the limits of its illuminated length, see Fig. 7 ▸. The beamlets are reflected and hit a fluorescence screen at the nominal focus distance. Their locations on the screen are recorded one after the other with a camera, and the evaluation over several shots gives the center of gravity for each beamlet. With this setup, the bender motors B_1_ and B_2_ are scanned until all three beamlets hit the screen at the same position. As only the centers of the beamlets are required to find the optimum bender settings, the method does not need an imaging system with a resolution better than the expected spot size, nor is it hindered by saturation effects in the camera or blooming in the screen. The drawback of this method is that the absolute size of the focus can only be determined down to the spatial resolution of the imaging system with all the given limitations, although the real spot size may be smaller.

**Figure 8:**
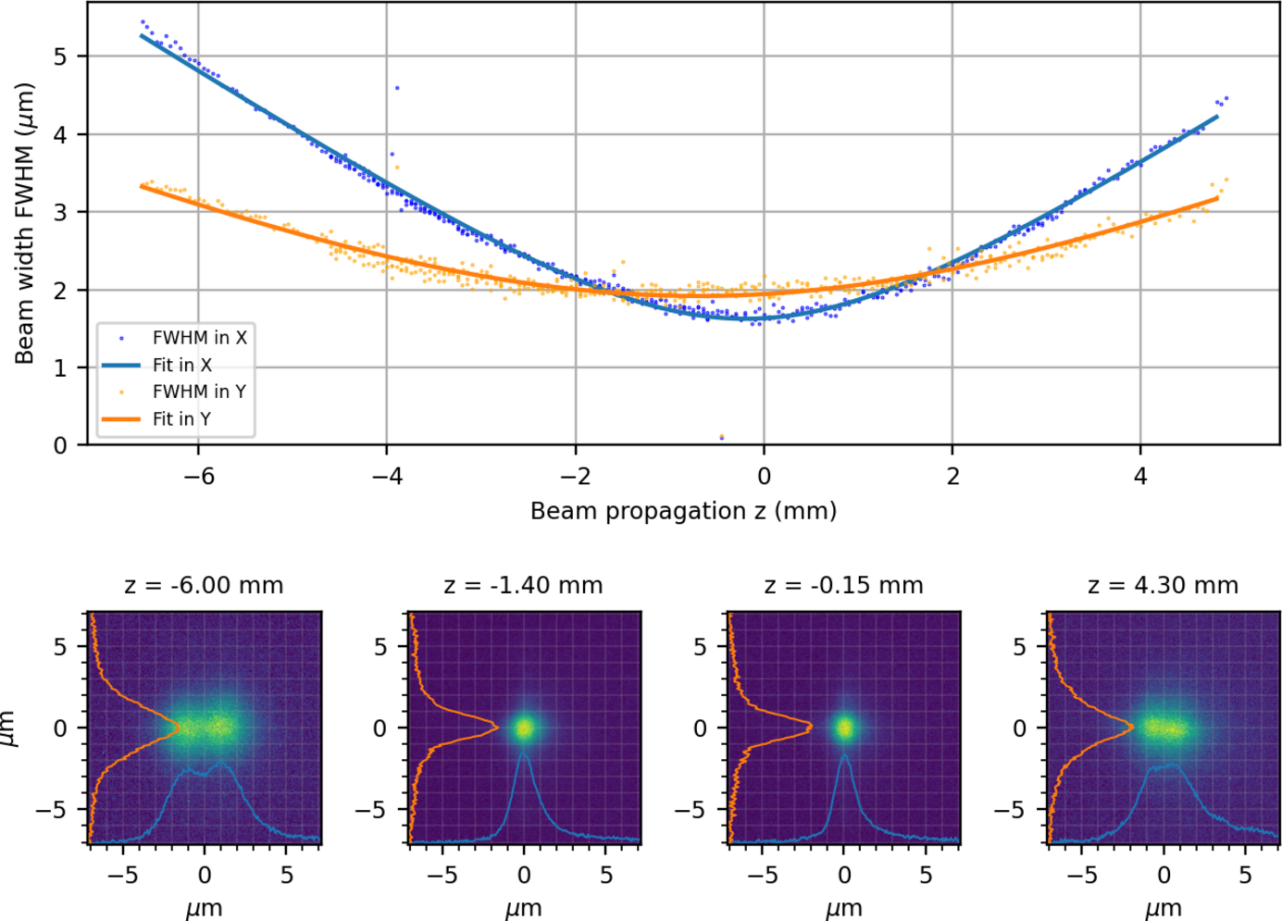
(Top) Focal image along the *X* and *Y* axis over a caustic scan along the X-ray propagation direction. The dots represent single-image acquisitions, and solid lines are based on Gaussian beam propagation in focus. (Bottom) Image of the X-ray focal spot at four different *Z* positions with projections of the beam intensity along the *X* and *Y* axes.

A caustic measurement of the focused beam was then performed, recording the focal spot of the X-rays by moving the vacuum chamber along the X-ray optical axis. Fig. 8 ▸(*a*) shows the fit of the beam waist along the *X* and *Y* axis versus the *Z* position (optical axis). The zero value represents the position where the X-rays were focused, showing the smallest size of 1.5 µm × 2 µm in the *X* and *Y* directions. When performing a caustic scan, it becomes apparent that the *Y* axis was focused 1.4 mm downstream of the *X* axis, indicating an astigmatic beam. To confirm the measured focal spot sizes, knife-edge scans yielded 1.45 µm and 1.7 µm FWHM for the *X* and *Y* axes. The imaging system overestimates the focal spot dimension. This could be attributed to saturation reasons, as the X-ray beam was not attenuated during the scan; hence, saturation could happen close to the smallest spot. Further, when reaching 1 µm focal spot, we reach the resolution limit of the system.

Despite this discrepancy, valuable information about the beam astigmatism, rough focal spot size and propagation could be easily extracted. The caustic measurement presented was performed within a 3 min scan. Furthermore, the system can be seamlessly integrated into experimental setups, allowing it to be inserted briefly for beam diagnostics and removed shortly after, causing only a few minutes of measurement interruption, as described by Sun *et al.* (2022 ▸).

The proposed imaging systems may adversely affect experimental outcomes in specialized operation modes. In a two-color operation, for example, two X-ray pulses with distinct photon energies are generated in separate undulator sections [see Prat *et al.* (2022 ▸) for details]. These pulses originate from source points separated by ∼20 m. Since a single pair of KB mirrors with 1:1 imaging capabilities is employed to focus both incoming beams, the focal points of the two pulses do not align at the same position along the optical axis (*Z*-axis).

**Figure 9:**
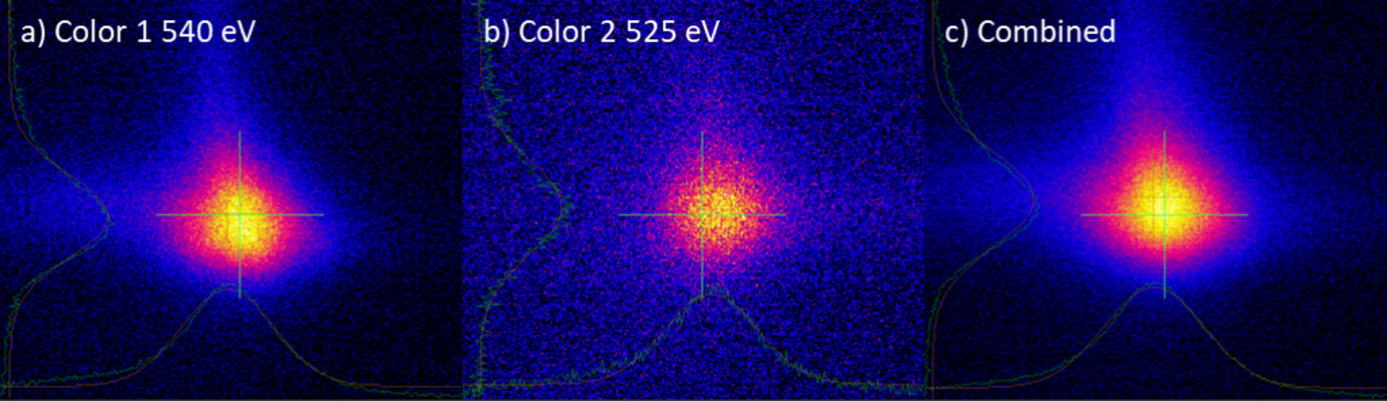
Images of two-color X-ray beams at the focus. (*a*) Color 1 at 540 eV photon energy generated in the first section of the undulators. (*b*) Color 2 at 520 eV photon energy generated in the second section of the undulators. (*c*) Both colors combined.

This effect is illustrated in Fig. 9 ▸, which displays the focal spots of the two colors. These images were obtained using the system described in Section 2.3. It is evident that, while the focal spot of color 2 is well focused, color 1 appears significantly larger. In this experiment, color 1 served as the excitation pulse and color 2 as the probe. Therefore, the system was optimized to produce a smaller focal spot for the probe (color 2), measured with a FWHM of 2.6 µm.

The beams may not propagate coaxially, resulting in focal points not overlapping spatially. Adjustments to the undulators and the electron beam trajectory were required to achieve the coaxial lasing of the two beams. This alignment was verified by imaging the focal spots of the individual colors at the sample position with micrometre-level spatial resolution, confirming that the beams were aligned coaxially and overlapped at the focal point.

### 3.4. Capabilities and opportunities

To demonstrate the capabilities of the proposed imaging system, we employ the setup described in Section 2.2 in combination with a transmission grating to generate high-frequency interference patterns. In this geometry, interference between the fundamental beam and its diffraction orders creates repetitive image replicas of the grating along the optical propagation axis (Yamada *et al.*, 2020 ▸; Seaberg *et al.*, 2019 ▸). A 2 µm-pitch transmission grating was placed 160 mm downstream of the X-ray focus, with the imaging system positioned 160 mm further downstream. The scintillator screen was aligned to the 33rd Talbot order, producing a magnified grating image with a 4 µm pitch.

This experimental approach leverages single grating Talbot interferometry (SGTI), a powerful wavefront sensing and phase imaging technique ideal for *in situ* diagnostics of X-ray beams (Yamada *et al.*, 2020 ▸; Seaberg *et al.*, 2019 ▸). SGTI exploits the Talbot effect, where the periodic grating self-images at specific Talbot distances, and enables precise phase and amplitude extraction from interference patterns. SGTI provides several significant advantages, such as accurate knowledge of the X-ray focus profile, the ability to operate out of focus to avoid damage to sensitive detectors, and the provision of single-shot information essential for capturing transient phenomena.

**Figure 10:**
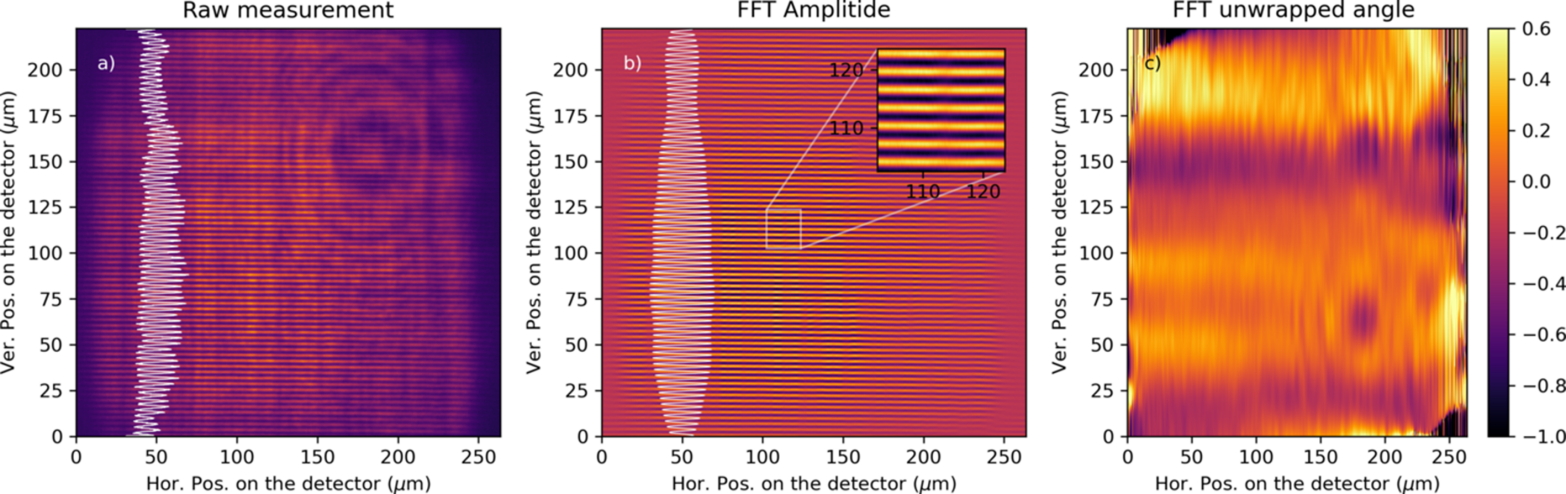
(*a*) Image generated by a single X-ray pulse using a 2 µm pitch grating. (*b*) The real part of the Fourier-filtered image is extracted, and the self-image of the grating is observed. (*c*) Phase of the image oscillatory pattern across the image with linear contribution removed. The color map represents the phase in radians.

Fig. 10 ▸(*a*) shows an image of a single X-ray pulse with 3 µJ at 1240 eV (1 nm). A circularly polarized X-ray beam was used to avoid third harmonic contamination. The white line is a projection along the image to the left, showing the contrast of the fringes. In this image, an Airy pattern due to a dust particle on the KB mirrors could be observed. This diffraction pattern interferes with the incoming X-ray beam, thus altering its wavefront. Considering the beam footprint, the power density is below 8 × 10^−2^ nJ µm^−2^, well within the linearity regime discussed in Section 3.2. This allows the measurement of the interference pattern without saturation and nonlinearity artifacts.

By employing Fourier filtering (Butterworth bandpass filter), the oscillatory pattern and its phase were isolated. The real part is presented in Fig. 10 ▸(*b*), with a zoomed-in section in the inset. Fig. 10 ▸(*c*) presents the unwrapped high-order phase of the image. The phase demonstrates the system’s sensitivity to wavefront distortions. The dust particle, acting as a landmark, can be readily observed in the phase, including the Airy disk pattern around it. A replica image in the phase can be observed, which indicates that the Talbot conditions were not met in this geometry.

**Figure 11:**
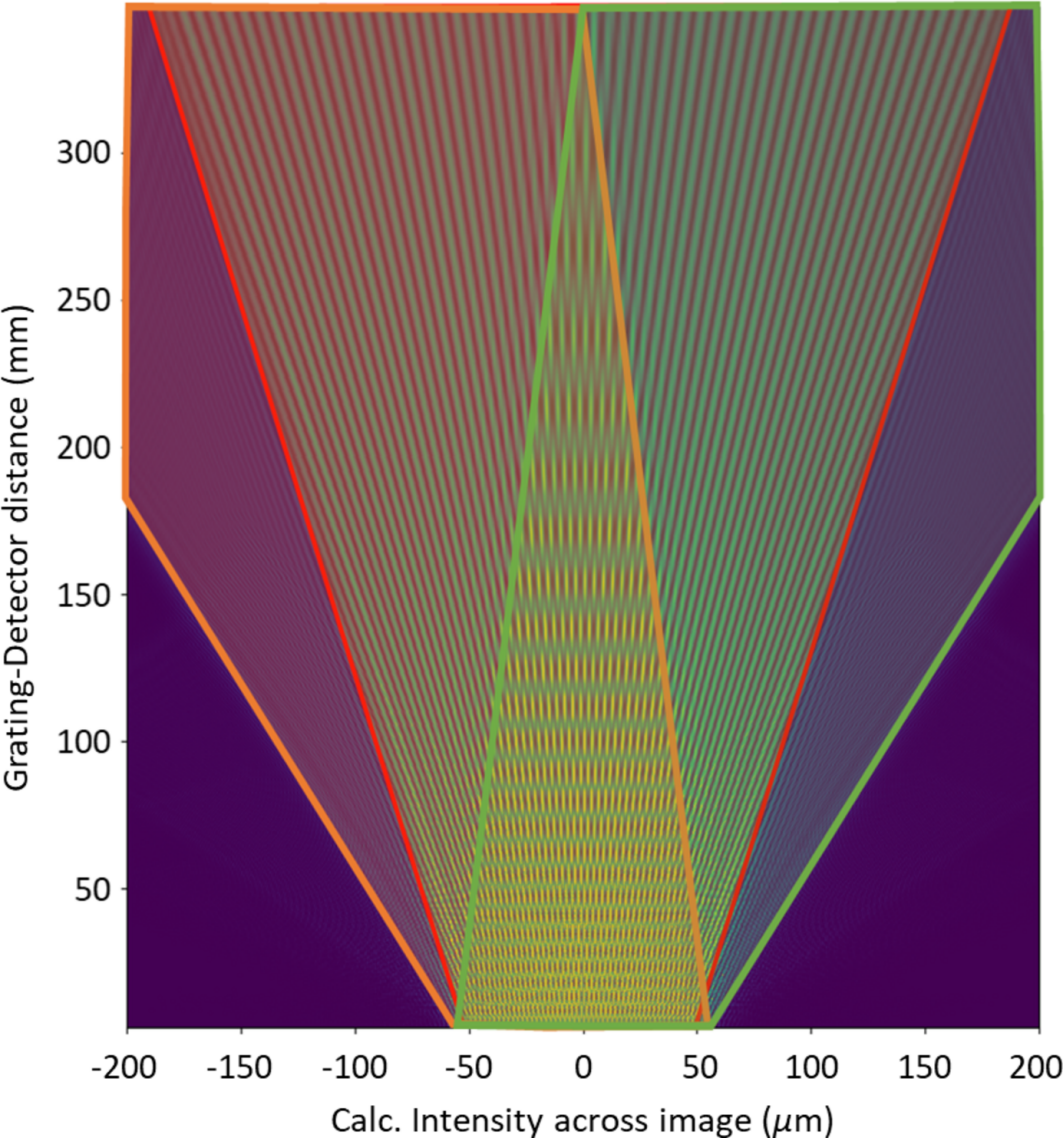
Wavefront propagation was simulated while incorporating experimental conditions. The fundamental, +1 and −1 diffraction order beams are outlined in red, green and orange, respectively. The central triangular region contains the Talbot carpet, which exhibits the Talbot self-imaging effect modulated along the optical axis. The interference patterns observed outside the Talbot carpet remain consistent and arise from the interaction between the fundamental beam and one of the diffraction orders.

To understand the source of the observed interference pattern, a wavefront propagation simulation was conducted using the LightPipes Python library, incorporating the experimental conditions (Vdovin & Van Fred, 2017 ▸). Fig. 11 ▸ illustrates the propagation along the optical axis between the 2 µm pitch grating and the detector, with distances varying from 30 mm to 350 mm. The fundamental beam (outlined in red) interacts with the +1 and −1 diffraction orders (outlined in green and orange, respectively). The Talbot carpet, centrally located and enclosed by these diffraction orders, exhibits periodic Talbot self-imaging as different orders are traversed. Beyond the Talbot carpet, interference between the fundamental and first-order beams is observed. At large grating–detector distances, half of the fundamental beam interferes with a portion of the first-order diffraction beams. Due to the inherent symmetry of the system, with +1 and −1 orders, the resulting interference pattern maintains symmetry in both intensity and phase.

**Figure 12:**
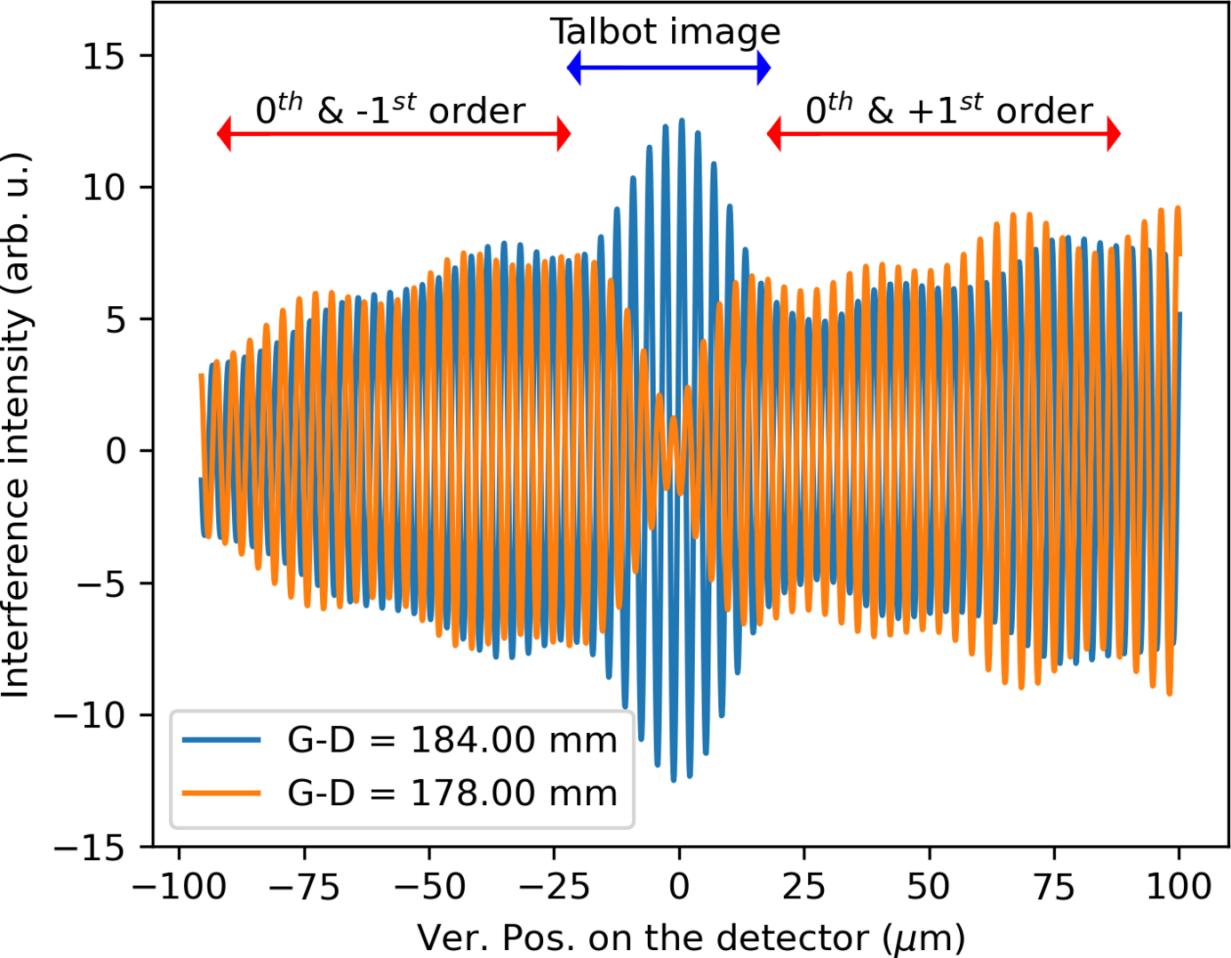
Fourier-filtered spectral interference patterns were measured at grating–detector distances of 178 mm and 184 mm. At 178 mm, the interference pattern appears split around the image center, indicating a position outside the Talbot plane. At 184 mm, corresponding to Talbot order 33, a central interference pattern is observed, with symmetric interference features surrounding it.

The simulation confirms what was measured in the experiment. The Fourier-filtered interference patterns at grating–detector distances of 178 mm and 184 mm are shown in Fig. 12 ▸. At 178 mm, a fully symmetric interference pattern is observed, characterized by a central minimum. At 184 mm, a pronounced central interference feature emerges, corresponding to the 33rd Talbot order. The experimental measurements closely align with the simulations, capturing both the interference patterns outside the Talbot carpet and the periodic appearance of the Talbot’s self-image. The interference patterns outside the Talbot carpet still hold substantial information about the beam divergence and partial phase.

Using the phase measurements taken at a focus–grating (FG) distance of 160 mm and a grating–detector (GD) distance of 145 mm, a focus reconstruction was performed along the vertical axis. To evaluate the accuracy and quality of this reconstructed focus, an additional measurement was conducted using a secondary detector positioned directly at the X-ray focal plane. This secondary detector was based on a commercially available off-the-shelf system, incorporating an Olympus objective with a NA of 0.9 (RMS60X-PFC).

Using this knowledge, we measured the interference pattern using the SGTI setup at an FG distance of 160 mm and a GD distance of 146 mm. This condition matches a Talbot self-image and the mirrored interference patterns around it. The complete wavefront characterization using SGTI involves determining both the amplitude and phase information of the wavefront by employing the first-order Fourier filtering discussed earlier. We note that the amplitude is directly proportional to the intensity profile of the beam, while the phase information is linked to the gradient of the wavefront. Together, these two components define the complex electric field of the X-ray beam, which we subsequently back-propagate to the focus. This back-propagation is vital for understanding how the beam will behave at the focus, enabling better optimization of experimental conditions and improving the overall diagnostics of XFEL optics (Seaberg *et al.*, 2019 ▸).

**Figure 13:**
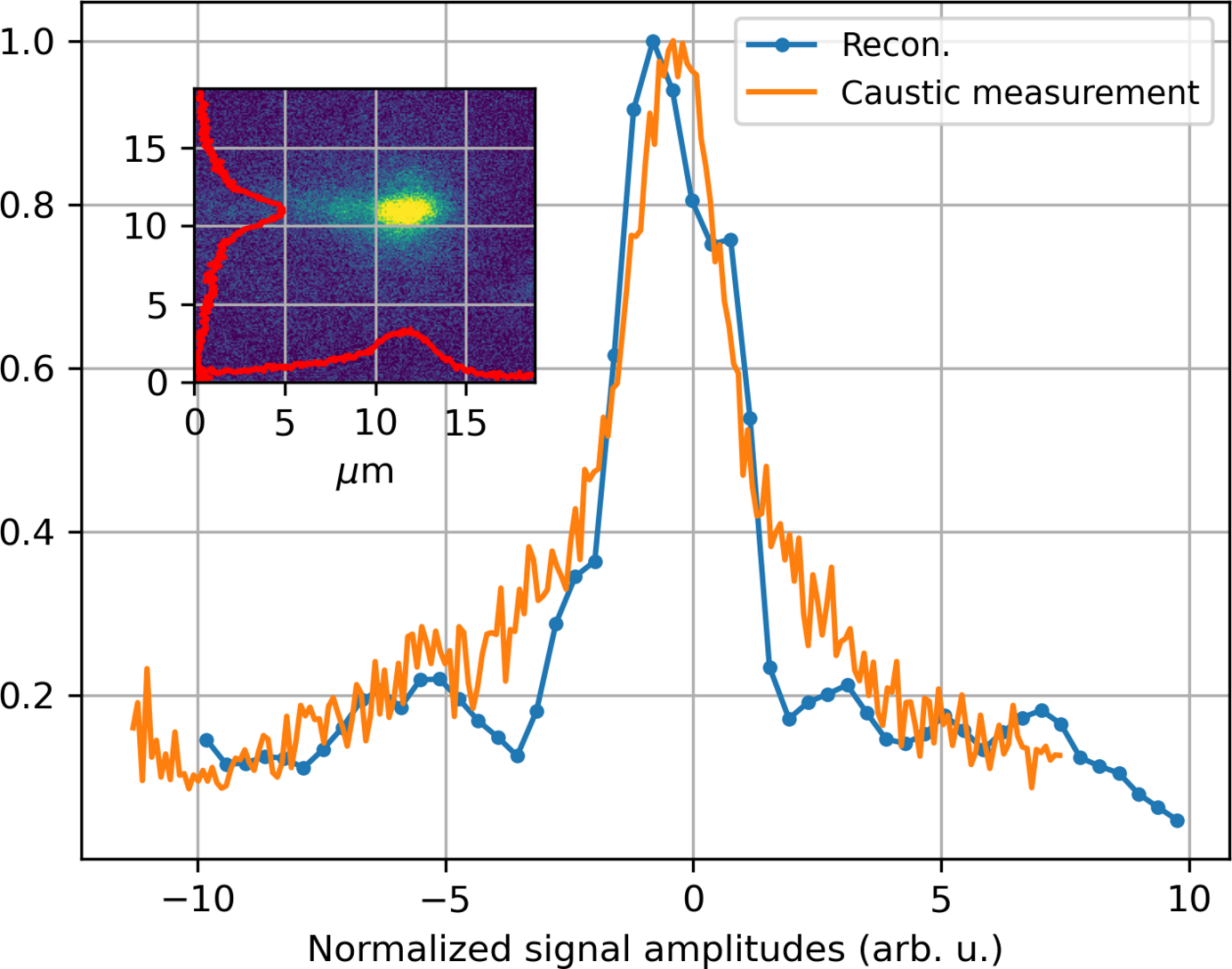
The blue curve represents the focus size determined through back-propagation using the measured phase and amplitude of the incoming X-ray beam. The orange curve depicts the vertical projection of the X-ray beam footprint measured at the focus position. The inset displays the beam footprint measured at the focus.

Fig. 13 ▸ displays the retrieved through-focus intensity profiles in the vertical projection, averaged from 1000 FEL shots measured at 1.24 keV (blue lines), compared with the typical through-focus lineout obtained from a scintillator screen (orange line). The beam caustic in the vertical direction shows agreement with the back-propagation, with an estimated 3.7 µm in the vertical direction. Compared with the caustic image, SGTI provides better contrast and enhanced details on the beam pedestals, likely due to the lower resolution of the imaging system and reduced contrast at the beam pedestals caused by a low signal-to-noise ratio at low intensities. This highlights the superiority of the SGTI method and its capability to capture critical details essential for characterizing X-rays and optimizing X-ray optics.

**Figure 14:**
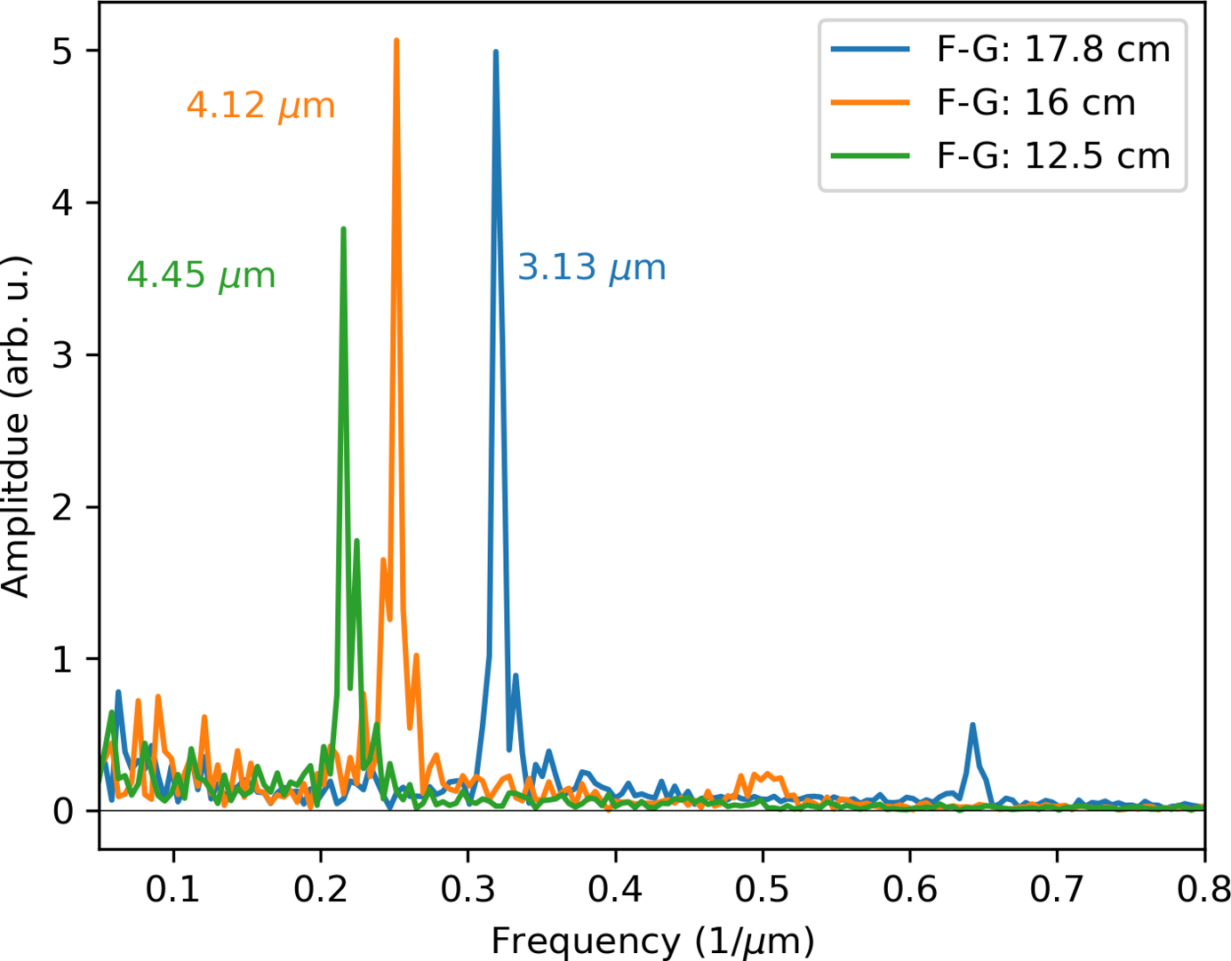
Fourier transforms of the images at three distinct beam focal points. The grating–detector distance was fixed to 15 cm, while the focus was moved along the optical axis to 12.5, 16 and 17.8 cm away from the grating.

By adjusting the focal plane of the X-rays relative to the grating–imaging system pair, the Talbot conditions remain consistent, yet the magnification of the self-image changes. In Fig. 14 ▸, we show the frequency components of the Fourier transform after moving the focal plane to three distances. For the starting condition, where the FG and GD distances are both equal to 160 mm, a magnification of 2× is expected, resulting in a 4 µm pitch in the grating self-image, which is observed in the frequency peak at 0.24 µm^−1^ (corresponding to 4.12 µm pitch). For FG distances larger or smaller than 16 cm, the frequency of the self-image shifts to 0.224 and 0.319 µm^−1^, respectively.

**Figure 15:**
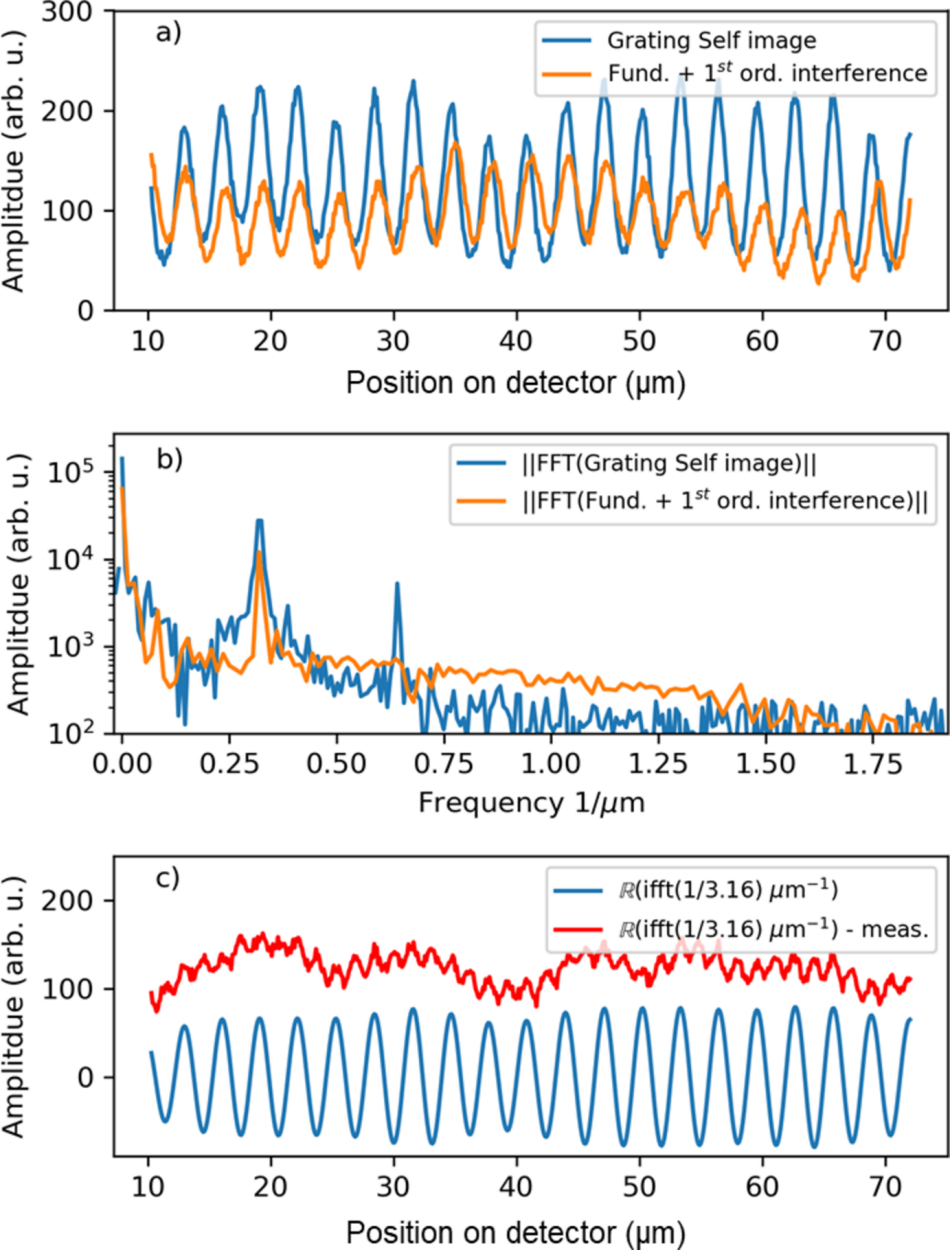
(*a*) A line-out over a width of 16.5 µm from the image in Fig. 10 ▸(*a*) is taken in the Talbot grating self-image area, and out of it. (*b*) A Fourier transform of both line-outs is presented. (*c*) The real part of the inverse Fourier transform of the low-frequency component at 3.13 µm^−1^ is presented in blue. The red curve is the residual between the low-frequency component and the measured line-out at the Talbot self-image.

A secondary peak at half the primary frequency is consistently observed in the system’s frequency spectra. Fig. 15 ▸(*a*) presents a horizontal axis projection of a 16 µm × 60 µm segment from a single X-ray pulse. The blue curve corresponds to the center of the image, where the grating self-image is present, while the orange curve represents the side of the image, where the fundamental beam interacts only with one of the diffraction orders. The Fourier transform of both curves is shown in Fig. 15 ▸(*b*) on logarithmic scale, revealing that the high-frequency oscillation appears exclusively in the Talbot self-image region and is absent elsewhere.

The observed oscillatory pattern in the Talbot grating self-image deviates from a purely sinusoidal form, which can be attributed to the grating’s unequal duty cycle. By subtracting the real part of the principal frequency component from the measured data, the high-frequency oscillatory component emerges in the residual, as shown in Fig. 15 ▸(*c*). This underscores the imaging system’s sensitivity and its capability to detect subtle variations caused by the grating’s uneven duty cycle.

## 4. Conclusion

This study introduced a series of UHV-compatible imaging systems capable of directly characterizing soft X-ray beam spatial modes with micrometre-level resolution. Performance evaluations demonstrated spatial resolutions on the order of 1 µm and sensitivities down to sub-nanojoule levels, enabling precise beam profile measurements and facilitating advanced experiments, such as the two-color mode presented.

To showcase the system’s capabilities, wavefront measurements were conducted using single-grating Talbot interferometry in a compact setup. The results demonstrated the system’s ability to resolve high-frequency modulations and perform wavefront measurements within an experimental footprint of less than 40 cm. The reconstructed focus via back-propagation exhibited excellent agreement with in-focus scintillator measurements. Additionally, the system successfully extracted sub-3 µm oscillations in the Talbot plane, resolving an oscillatory pattern resulting from the grating’s ≠0.5 duty cycle.

The versatility of these imaging systems ensures their compatibility with a wide range of experimental setups, emphasizing their adaptability for various applications in X-ray science.
